# A UK-wide survey of Balint, support groups and psychotherapy training opportunities for SAS (Specialty Doctors and Associate Specialists) Psychiatrists

**DOI:** 10.1192/bjo.2021.197

**Published:** 2021-06-18

**Authors:** Alina Vaida, Masud Awal

**Affiliations:** Birmingham and Solihull Mental Health NHS Foundation Trust

## Abstract

**Aims:**

To investigate SAS Psychiatrists’ opportunities for Balint-type, support groups and psychotherapy training opportunities nationwide, for which there is a lack of existing literature or established framework.

**Method:**

An online questionnaire was sent to UK-wide SAS psychiatry doctors with the support of the RCPsych Speciality Doctors and Associate Specialist Psychiatrists Committee (SASC). The survey enquired about location, work experience, future plans, Balint-type groups, psychotherapy opportunities and support.

**Result:**

122 doctors completed the questionnaire, estimated to constitute approximately 8% of SAS psychiatry posts (or more if considering all vacancies), based on the RCPsych Census (2015), from across all UK nations.Time spent in an SAS role varied widely between months (10%) to over 20 years (5%), with the median and mode being 8–12 years (25%). Regarding future career plans 61% responded that they would be considering either the Certificate of Eligibility for Specialist Registration (CESR) route, or applying for future training or both.

24% reported being part of a Balint-type group whilst almost double this number (47%) said they would be interested to join but none were available. 31% were part of a reflective practice or support group whilst 44% reported that they were interested in joining but none were available. Only 7% said that they were not participating or not interested in either a Balint group or a reflective group. Free-response comments suggested these opportunities were usually reserved for trainees and service commitments prevented attendance.

76% of respondents reported access to an SAS Tutor, but only 21% confirmed access to a psychotherapy tutor.

Half of respondents indicated they did not have access to information and guidance they needed regarding accessing psychotherapy opportunities, with only 27% thinking they did.

24% reported managing to gain experience in at least one psychotherapeutic modality, 44% of whom received medical psychotherapist supervision; whilst 13% said they did not intend to pursue this.

**Conclusion:**

The results highlight that interest in joining Balint and reflective support groups significantly exceeds local provision. As these groups are not mandatory requirements for CESR application, the interest expressed (including amongst those reporting to be SAS by choice) suggests that SAS Psychiatrists value these opportunities for their recognised professional developmental and clinical benefits; these include peer support, understanding doctor-patient interactions and having a space to reflect on the emotional impact of clinical work. Trusts should consider supporting SAS doctors wishing to join new or existing Balint-type or other supportive reflective clinician groups.

